# Ophthalmological screening guidelines for individuals with Osteogenesis Imperfecta: a scoping review

**DOI:** 10.1186/s13023-024-03285-9

**Published:** 2024-08-30

**Authors:** Sarah Moussa, Jasmine Rocci, Reggie Hamdy, Jakob Grauslund, Marie-Louise Lyster, Argerie Tsimicalis

**Affiliations:** 1 Faculty of Medicine and Health Sciences, McGill University, Montréal, Canada; 2 Shriners Hospitals for Children®-Canada, 1003, boulevard Décarie, Montréal, QC H4A 0A9 Canada; 3 Department of Ophthalmology, Odense University Hospital, Odense, Denmark; 4 Department of Clinical Research, University of Southern Denmark, Odense, Denmark; 5 Department of Ophthalmology, Vestfold Hospital Trust, Tønsberg, Norway; 6 Department of Endocrinology, Odense University Hospital, Odense, Denmark; 7 Faculty of Medicine and Health Sciences, Ingram School of Nursing, 680 Sherbrooke Street West, H3A 2M7 Montreal, QC Canada

**Keywords:** Osteogenesis imperfecta, Brittle bone disease, Ophthalmology, Screening, Prevention, Review, Knowledge synthesis, Guidelines, Ocular manifestations, Optometry, Eye disease, Eye manifestations

## Abstract

**Background:**

Osteogenesis imperfecta (OI) is a connective tissue disorder in which the Type 1 collagen is defective. The eye is a structure rich in collagen Type 1 and is heavily impacted by the disease. Many vision-threatening eye diseases have been associated with OI. The onset of these diseases also tend to occur at an earlier age in individuals with OI. Despite the research on these risks, appropriate ophthalmological screening or care guidelines for individuals with OI remain unknown. As such, the purpose of this scoping review was to explore and describe existing ophthalmological screening and care guidelines to orient OI patient care.

**Main body:**

A scoping review based on the Joanna Briggs Institute (JBI) methodology was conducted. A search of databases (PubMed and Medline) was completed in consultation with a research librarian. A total of 256 studies were imported for screening. Primary sources matching the inclusion and exclusion criteria were screened, extracted, and analyzed using Covidence.

**Conclusion:**

A total of 12 primary articles met inclusion and exclusion criteria, containing case reports, case series and cohort studies. Despite the risk of blindness associated with the consequences of OI on the eye, the primary literature fails to provide detailed screening and care guidelines aimed at identifying disease early. We provide general recommendations based on the review findings to guide the ophthalmological care of patients with OI and call upon the experts to convene globally to create screening guidelines. Further investigations of ophthalmological screening are warranted to limit these vision-threatening risks with early detection and treatment. Standardized ophthalmological screening guidelines for OI remain an area for research.

**Supplementary Information:**

The online version contains supplementary material available at 10.1186/s13023-024-03285-9.

## Background

Osteogenesis imperfecta (OI), also referred to as brittle bone disease, is a rare connective tissue hereditary disorder affecting collagen formation [1]. This predominantly autosomal dominant disease is characterized by bone fragility, multiple fractures, and subsequent skeletal deformity [1]. Other notable findings associated with OI are hearing loss, cardiovascular disease, dentinogenesis imperfecta, and blue sclera [2]. The reported prevalence is 1 in 15,000–20,000 births [1]. Most cases (90%) are due to a mutation in the COL1A1 or COL1A2 genes which encode for type I collagen [1]. OI is further subdivided: type I OI is caused by a quantitative defect in collagen, while types II-IV OI are caused by qualitative structural defects [1]. Collagen Type I is the major structural protein in the eye [1]. Collagen is a crucial structural component of the cornea and sclera [3]. The cornea provides most of the refractive power essential for good visual acuity whereas the sclera maintains the integrity of the eye with its strength and structure [3]. Collagen type I is also found in the retinal vessels and the Bruch’s membrane, which supports the retina [3]. Collagen type I is a structural component of the trabecular meshwork of the eye, which is responsible for the aqueous humour flow [3]. The optic nerve is also surrounded by collagen type I and is thus essential for its function (3). Hence, collagen type I plays a crucial role as a major structural element in the eye. However, there is limited understanding of how OI affects the eye. A large register-based cohort study by Lyster et al. (2022) of 907 OI patients compared to a reference cohort found a higher incidence of cataracts and glaucoma in OI patients. Individuals with OI were also at an increased risk of refractive disorders, vitreous hemorrhage, retinal detachment or ruptures, other retinal diseases (such as retinopathy, retinal hemorrhage or degeneration), as well as optic nerve disorders. A systematic review by Treurniet et al. (2022) highlights that practically every anatomical portion of the eye can be impacted by OI. Despite the potential vision-threatening complications linked to OI, little is known about appropriate ophthalmological care guidelines and screening for patients with OI. The primary aim of this scoping review was to map existing screening recommendations and patient care guidelines provided in the ophthalmology literature for individuals with OI, setting the stage for future consensus planning and screening recommendations by global experts.

## Methods

This scoping review was guided by the Joanna Briggs Institute (JBI) scoping review methodology and the PRISMA scoping review checklist [4, 5]. No review protocol was registered for this study.

### Information sources and search strategy

A review of the major medical databases, including Medline (OVID) and PubMed, were conducted in consultation with a research librarian. For each database, specific search terms were used to address the concept of OI and ocular manifestations. The first concept of OI also included its synonym, “brittle bone disease”. The second concept focused on ophthalmology-specific terms and ocular manifestations that were associated in the literature for keywords, Medical Subject Headings (MeSH) terms, and exploded commands [2, 6] (Supplemental Table 1). Terms included: ophthalmology OR optometry OR eye disease OR eye manifestations OR corneal diseases OR cataract OR refractive errors OR glaucoma OR vitreous hemorrhage OR retinal detachment OR optic nerve diseases OR retinal diseases. The initial literature search was conducted in August 2022, and a re-run of the search was completed in June and October 2023 for any new articles. In addition to these database searches, a snowball technique inspecting the references of the final included studies was completed to identify further qualifying items.

### Eligibility criteria

Inclusion criteria include all primary literature reporting on the eyes of individuals of all ages with OI in English or French from 1980 onwards. Language criteria reflect the primary languages of the research team. Data were considered from 1980 onwards to ensure that the medical recommendations pertaining to screening remain relevant in the field of ophthalmology. Exclusion criteria included review articles and literature that did not propose specific screening tests, patient care guidelines or recommendations.

### Data charting and data items

Two reviewers independently assessed each article according to the pre-established eligibility criteria via the Covidence Software (SaaS enterprises, United States). The initial screen included titles and abstracts, with a narrowed screening for full-text articles. Conflicts were resolved during one-on-one meetings through consensual discussions. One reviewer extracted the data of the eligibility articles, which included: title, publication year, geographical location, study setting, purpose, study design, sample size, sample description, types of OI studied and the screening tools, guidelines or recommendations proposed. Data were analyzed descriptively using Excel (Microsoft, United States), synthesized narratively, and portrayed graphically.

## Results

### Search results

**Fig. 1:**
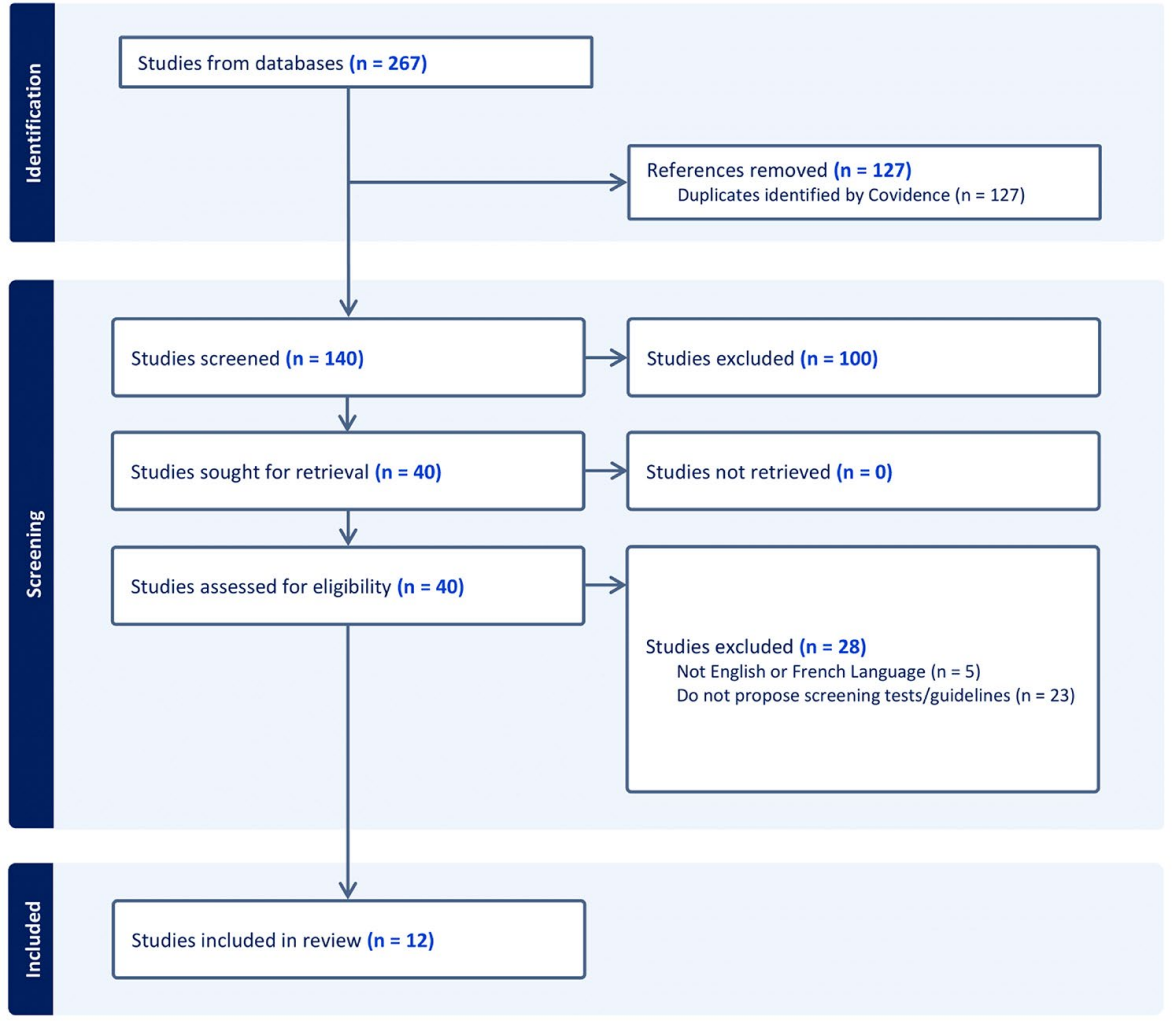
PRISMA flow diagram [5]

A total of 139 unique articles were retrieved from PubMed and Medline (OVID) (Fig. 1). Screening of the title and abstract, followed by full-text screening, yielded a total of 12 articles which met the eligibility criteria. No additional articles were successfully identified via the references snowball technique. Twenty-three articles (58%) were rejected as the authors did not propose any screening or patient care recommendations derived from their findings. The PRISMA flow diagram, which provides details about the selection process, is shown in Fig. 1.

### Characteristics of studies

**Fig. 2:**
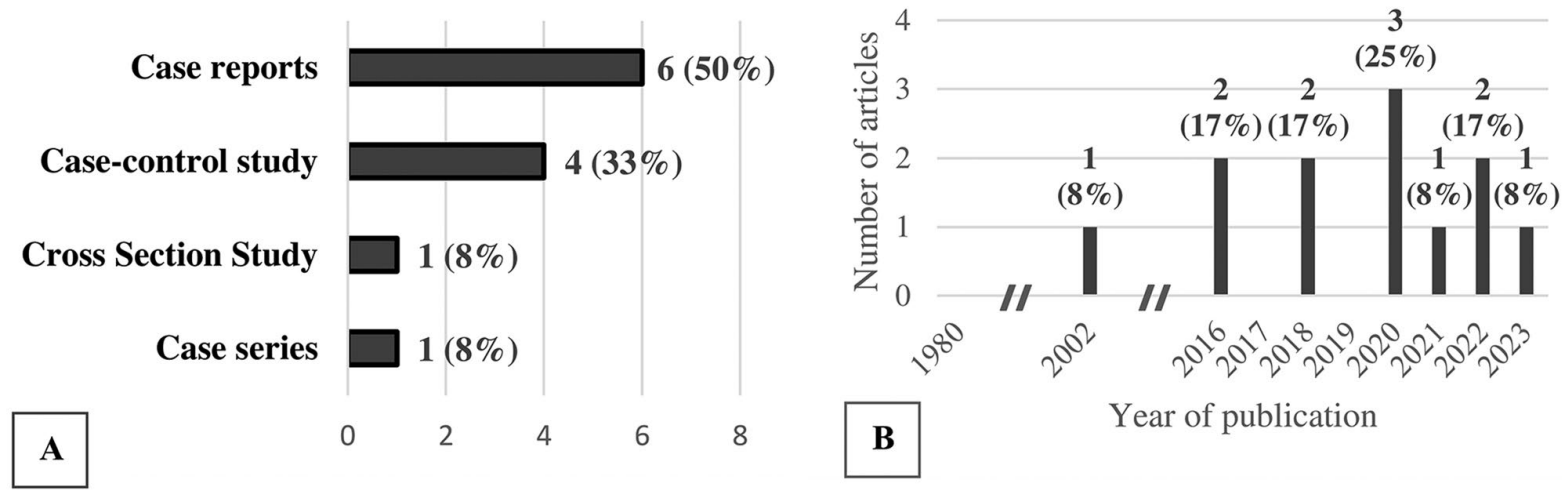
Type of data sources (**2a**) and publication dates (**2b**) of available ophthalmological recommendations for patients with OI (*n* = 12)

Cumulatively, 1,084 individuals with OI were included for study [6–17]. Most final articles were case reports or case-series (*n* = 6, 50%, *n* = 15/1084 = < 0.01%, Fig. 2a) [7, 8, 14–17], followed by case-control studies (*n* = 4, 33%, *n* = 984/1084 = 91%, Fig. 2a) [6, 9, 11, 13]. One cross sectional study design (*n* = 1, 8%, *n* = 85/1084 = 0.08%, Fig. 2a) [12] and one case series were found (*n* = 1, 8%, *n* = 8/1084, < 0.01%) [10]. Most articles were published in the last seven years (*n* = 11, 92%, n = Fig. 2b) [6–10, 12–17]. The majority of recommendations originate from studies conducted in Western Europe (*n* = 5, 42%, *n* = 954/1084 = 88%) [6, 8–10, 12] and Turkey (*n* = 4, 33%, *n* = 62/1084 = 6%) [7, 11, 13, 17] (Table 1). The majority of the recommendations are derived from high-income countries (*n* = 7, 58%, *n* = 1040/1084 = 95%) (Table 1) [6, 8–10, 12, 14, 15], and the rest come from upper-middle-income countries (*n* = 5, 42%, *n* = 63/1084 = 5%) (Table 1) [7, 11, 13, 16, 18]. A large portion of the articles had adults only as participants (*n* = 6, 50%, 1033/1084 = 95%) [6–9, 12, 16]. Some articles only included children (*n* = 4, 33%, 26/1084 = 2%) [11, 14, 15, 17], and others included a mix of both children and adults (*n* = 2, 17%, 25/1084 = 2%) [10, 13].

**Table 1:** Study characteristics and ocular screening guidelines and recommendations for OI patients derived from 12 studies (*n* = 1,084)

Authors	Publication year	Geographical Location & Income	Sample Size	Sample description	Type(s) of OI studied	Screening Guideline or Recommendation
Oksan Alpogan [7]	2022	Turkey/HMIC	2	• 58-year-old man and his 31-year-old daughter	OI Type I	• Screen for glaucoma may be indicative
Bellanca, et al. [8]	2020	Italy/HIC	1	• 28-year-old male	OI Type I	• Assess and monitor retinal abnormalities
Correia Barão, et al. [9]	2023	Portugal/HIC	37 (74)	• 37 adult OI patients • 37 age-matched controls		• Screen for keratoconus via tomography may be warranted due to significant differences in several tomographic keratoconus indices
Emer Doolan & Colm O’Brien [10]	2021	United Kingdom/HIC	8	• Sex: 7 females, 1 male • Age: early 50s to child age.	OI Type I	• Use corneal hysteresis for those at risk of glaucoma.
Evereklioglu, et al. [11]	2002	Turkey/HMIC	23 (38)	• 23 children with OI (13 boys, 10 girls) • 15 age, sex, and refraction-matched control subject (8 boys, 7 girls)	OI Type I OI Type IV	• Integrate central corneal thickness (CCT) measurements in all ocular examinations • Recommends CCT measurement when developing an intraocular pressure (IOP) protocol.
Hald, et al. [12]	2018	Denmark/HIC	85	• Danish adult patients 19–78 years old.	OI Type I OI Type III OI Type IV	• Consider CCT in the diagnostic process of individuals with probable OI.
Keleş, et al. [13]	2020	Turkey/MIC	17 (36)	• 17 OI patients (6–22 years old) • 19 age-matched control subjects	OI Type I OI Type II OI Type III OI Type IV	• Perform corneal analysis including CCT for monitoring
Kyo Oh, et al. [14]	2016	South Korea/HIC	1	• 18-month-old male	OI type I	•Screen pediatric population by ophthalmologists with CCT measurement
Lyster, et al. [6]	2022	Denmark/HIC	907 (5442)	• 907 OI patients (493 women) • 4535 persons from the reference population (2465 women)	Not reported	• Offer examinations by ophthalmologists with specific attention to risks associated with OI to detect vision-threatening ocular diseases at an early stage.
Scollo et al. [15]	2018	United Kingdom/HIC	1	• 9-year-old boy	OI Type VIII	• Conduct genetic analysis to guide ophthalmology monitoring programs and inform prophylactic treatment to reduce the probability of retinal detachment.
Todeschini de Souza, et al. [16]	2021	Brazil/HMIC	1	• 28-year-old female	OI Type VIII	• Offer fundoscopic examination
Yekta Sendul, et al. [17]	2016	Turkey/HMIC	1	• 12-year-old girl	Not reported	• Conduct annual ophthalmological eye examination to help identify papilledema.

HIC: High income country, HMIC: High-middle income country

### Ophthalmological screening

**Fig. 3:**
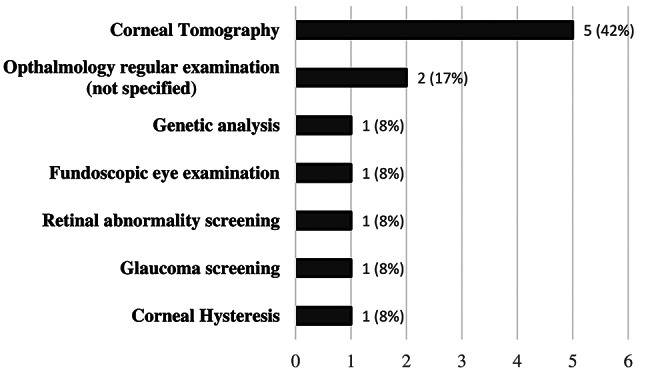
Summary of the screening recommendations for OI patients

Several authors recommended using corneal tomography for OI patients (*n* = 5, 42%, Fig. 3) [9, 11–14]. This 3-D imaging modality allows clinicians to assess corneal thickness distribution, specifically the central corneal thickness (CCT) [19]. Other recommendations include general “regular examination” by an ophthalmologist (*n* = 2, 17%) [6, 14], fundoscopic examinations (*n* = 1, 8%) [16], corneal hysteresis measurement (*n* = 1, 8%) [10], glaucoma screening (*n* = 1, 8%) [7] and retinal abnormality screening (*n* = 1, 8%) [8]. The frequency of these examinations was not specified by the authors. Scollo et al. (2018) recommended using genetic testing as a marker for retinal abnormality risk in their case report and, as such, used for screening and identifying high-risk OI patients. (*n* = 1, 8%, Table 1) [15].

## Discussion

The purpose of this scoping study was to explore and map the ocular screening guideline and patient care recommendations for individuals with OI. Classic criteria for appropriate use of screening have been established in 1968 by the World Health Organization (WHO) following publication by Wilson and Junger [20]. Ophthalmological screening of OI patients fit all ten criteria [20]. The eye-related issues associated with Osteogenesis Imperfecta (OI) represent a significant health concern. There are effective treatments accessible, and they can be provided within different facilities. Additionally, appropriate tests and examinations can be conducted during latent or early symptomatic stages. These tests are non-invasive, thus can be well accepted by the OI population, and be a part of a continuous process with clear policies on treatment. Given the high financial burden of vision loss, the cost-finding is well balanced. Finally, the natural history of OI and the pathophysiology of its associated ocular complications are adequately understood. To the best of our knowledge, this is the first study attempting to explore screening recommendations provided in the field of ophthalmology in the context of OI care. This scoping review identified 12 studies, primarily case reports and case control studies, which proposed ophthalmological screening tests, guidelines, or patient care recommendations for patients with OI.

The cornea is rich in collagen type I and, as such, is impacted in patients with OI [2]. Corneal tomography allows clinicians to evaluate the thickness of the cornea, also known as corneal pachymetry, thus allowing them to track disease progression [21]. Previous literature suggests that individuals with OI, especially type I, have a significantly lower central corneal thickness (CCT) than the average population [2]. Keratoconus, a vision-threatening complication, is one of the diseases which can occur due to a progressive thinning of the cornea [22]. A recent case-control study with 37 OI patients and age-matched controls highlighted that tomographic keratoconus indices are more frequent in this population (60%) [9]. An additional complication of thinning of the cornea or sclera can be globe ruptures following minor traumas due to the increased fragility of these structures [2]. A globe rupture may result from minor eye rubbing, especially in pediatric populations [2]. The cornea is also a major source of refractive power in the eye, and alteration in its structure or thickness can also result in changes in visual acuity, most notably myopia in OI patients (2). Five of the twelve studies (42%) included in this scoping review recommended corneal tomography for individuals with OI as a screening tool [9, 11–14].

Oksan Alpogan (2022) recommends glaucoma screening [7]. A low CCT is also known to be linked to higher open-angle glaucoma [4]. A lower CCT can also result in an underestimation of intraocular pressure (IOP) measurement, thus making glaucoma screening and management more difficult [23]. Particular attention can be given to other aspects of the examination for glaucoma, such as optic disc visualization, cup-to-disc ratio and visual field testing [2]. Doolan and O’Brien (2021) recommend evaluating the biomechanical property of the cornea via corneal hysteresis [10]. Corneal hysteresis is a measurement of the ability of the cornea to absorb and dissipate forces, thus reflecting its elastic properties [24]. A low corneal hysteresis has been linked to a greater risk of glaucoma [24]. Individuals with OI mainly have low collagen type I, which can result in disturbance in the trabecular meshwork architecture, thus resulting in poor drainage of the aqueous fluid and subsequent higher IOP [25].

De Souza et al. (2021) recommend fundoscopic examination [16] and Bellanca et al. (2020) recommend retinal abnormality screening [8]. The retina is also composed of collagen type I and is thus susceptible to having the integrity of its structure at risk due to OI [2]. This may result in higher risks of retinal tears, detachment, or hemorrhages secondary to minor trauma [2]. Scollo et al. (2018) recommend was to use genetic analysis to stratify risk for retinal detachment [15].

Finally, regular ophthalmology examinations were recommended for OI patients (*n* = 2). A previous systematic review by Treurniet et al. (2022) on ocular manifestations of OI reported that there are no major indications for annual check-ups with ophthalmologists in the absence of symptoms [2]. More research is needed with the collaboration of experts in the field to help guide OI care and set formalized guidelines.

### Ophthalmology care in OI

Interdisciplinary care for individuals with OI is a necessity which has shown better patient outcomes and better patient satisfaction [26]. Ophthalmology is a highly specialized field in medicine and surgery, with access to care varying vastly from region to region, with some areas where it is highly accessible and others less so. Other physicians and professionals rely on available guidelines and expert opinion for referral and subsequent follow-up. Only recently has there been an increase in awareness and data on the various effects of OI on ocular health and the many complications that may arise [2, 6]. With these findings in mind, healthcare organizations and teams must act as patient advocates to ensure appropriate follow-up for optimal ocular health and, ultimately preservation of vision. In our current Canadian pediatric institutions, no clinical directives are in place to refer OI patients to an ophthalmologist unless there are acute ocular complaints shared with the medical team. Instead, patients are encouraged to seek optometry visits which are free-of-charge in our health systems until the age of 17 at their discretion. Subsequently, vision care in the adult system is not covered for most unless ophthalmology is consulted. In our European institutions, OI patients are encouraged to seek general ophthalmology care as part of their initial care plan. In light of the recent evidence on early risks of OI on vision and the recommendations postulated in this review, we suggest that, until more definitive guidelines are established, all OI patients be referred initially to an ophthalmologist who may be able to conduct a personalized risk assessment and guide patients to an individualized screening and prevention protocol based on their initial clinical evaluation. Future research should focus on stratifying the ocular risk by sub-types of OI given their vast clinical presentation, using age-specific risks stratification in screening protocols and assessing the methods and instrumentality of screening, cost-effectiveness and frequency of required screening with a focus on the accessibility of resources in which clinicians practice in (high income vs. low income). Future research should also assess the impact of positive family history of ocular complications and propose knowledge translation strategies to improve patient education with clear emergency protocols and continuing education opportunities for the interprofessional health care team.

### Limitations and future directions

This scoping review has some limitations, given the paucity of literature identified, and no external consensus sought. A grey literature search to retrieve blogs, conference proceedings, or thesis works was not conducted. The eligibility was restricted to articles published in English or French with five articles (4 case reports and 1 cross-sectional study), published in 1995, 2012 and 2023 were excluded due to language barriers [27–31]. Critical appraisal of the articles was not completed, given the nature of scoping reviews, and as such, no comments on the quality of the extracted recommendations can be provided thus limiting the depth of the analysis and the strength of the recommendations. Inconsistency in reporting of OI sub-types limited sub-group analyses warranting future reporting of sample to include OI types and other key demographic characteristics to describe the diverse patient population. There was significant heterogeneity in ages, and as such, no specific age-specific recommendations can be postulated for children or older people. In addition, the demographic diversity of patients as they pertain to race were not explicitly documented in the studies included [32]. All evidence is derived from high or middle-to-high-income resource settings. Recommendations related to fundoscopic examination, genetic analysis, retinal abnormality screening, glaucoma screening and corneal hysteresis were derived from case reports or case series [7, 8, 10, 15, 16]. Nevertheless, these findings serve as a first step towards building global discussion and consensus and paving a way forward for multi-site, rigorously conducted, international studies to develop and evaluate guidelines including in high and low-resource settings.

## Conclusion

Despite the risk associated with the consequences of OI on the eye, the primary literature fails to provide detailed and robust screening guidelines aimed at identifying disease early. It is important to further investigate ophthalmological screening opportunities for individuals with OI, thus limiting these vision-threatening risks with early detection and treatment. Standardized ophthalmological screening and patient care guidelines for OI remain an area for research and remains imperative to convene the global community to create consensus guidelines. Most articles were recently published in Europe. This would favour the creation of a European coalition of ophthalmologists and eye professionals to create clinical recommendations and guidelines based on expert opinion.

### Electronic Supplementary Material

Below is the link to the electronic supplementary material.

Supplementary Material 1

## Data Availability

All data generated or analyzed during this study are included in this published article [and its supplementary information files].

## Abbreviations

OI: Osteogenesis Imperfecta
HIC: High income country
MHIC: Middle high-income country
CCT: Central Corneal Thickness
IOP: Intraocular pressure
