# Supplementary material for: Ophthalmological screening guidelines for individuals with Osteogenesis Imperfecta: a scoping review
Source: Orphanet J Rare Dis. 2024 Aug 30;19:316. doi: 10.1186/s13023-024-03285-9 (PMC11363591; doi:10.1186/s13023-024-03285-9)
Supplement: Supplementary file 1 — Supplementary Material 1 [file 13023_2024_3285_MOESM1_ESM.docx]

# SUPPLEMENTAL FILES

**Supplemental Table 1– Search Query used databases (n = 2)**

| **Database** | **Search Query** |
| --- | --- |
| **PubMed** | ("Osteogenesis Imperfecta"[Mesh] OR "Brittle bone disease" OR "Osteogenesis Imperfecta") AND ("Ophthalmology"[Mesh] OR "Optometry"[Mesh] OR "Eye Diseases"[Mesh] or "Eye Manifestations"[Mesh] OR "Corneal Diseases"[Mesh] OR "Cataract"[Mesh] OR "Refractive Errors"[Mesh] OR "Glaucoma"[Mesh] OR "Vitreous Hemorrhage"[Mesh] OR "Retinal Detachment"[Mesh] OR "Optic Nerve Diseases"[Mesh] OR "Retinal Diseases"[Mesh] OR Ophthalmolog* or Optometr* OR "Eye disease*" or "Eye manifestation*" or "ocular manifestation*" OR "corneal disease*" OR "cataract" OR "Refractive error*" OR "Glaucoma" OR "Vitreous Hemorrhage" OR "Retinal Detachment" OR "Retinal rupture" OR "Optic nerve disease*" OR "Retinal disease*")  Filters: from 1980 – 2023 |
| **Medline (OVID)** | ((exp Osteogenesis Imperfecta/) OR (Osteogenesis Imperfecta) OR (Brittle bone disease)) AND ((exp Ophthalmology/) OR (exp Optometry/) OR (exp Eye Diseases/) OR (exp Eye Manifestations/) OR (exp Corneal Diseases/) OR (exp Cataract/) OR (exp Refractive Errors/) OR (exp Glaucoma/) OR (exp Vitreous Hemorrhage/) OR (exp Retinal Detachment/) OR (exp Optic Nerve Diseases/) OR (exp Retinal Diseases/) OR (Ophthalmolog*) or (Optometr*) OR (Eye disease*) or (Eye manifestation*) or (ocular manifestation*) OR (corneal disease*) OR (cataract) OR (Refractive error*) OR (Glaucoma) OR (Vitreous Hemorrhage) OR (Retinal Detachment) OR (Retinal rupture) OR (Optic nerve disease*) OR (Retinal disease*))  limit 6 to (yr="1980 -Current") |
